# A *Mycobacterium tuberculosis* Specific IgG3 Signature of Recurrent Tuberculosis

**DOI:** 10.3389/fimmu.2021.729186

**Published:** 2021-09-22

**Authors:** Stephanie Fischinger, Deniz Cizmeci, Sally Shin, Leela Davies, Patricia S. Grace, Aida Sivro, Nonhlanhla Yende-Zuma, Hendrik Streeck, Sarah M. Fortune, Douglas A. Lauffenburger, Kogieleum Naidoo, Galit Alter

**Affiliations:** ^1^Ragon Institute of MGH, MIT and Harvard, Boston, MA, United States; ^2^University of Duisburg-Essen, Essen, Germany; ^3^Department of Biological Engineering, Massachusetts Institute of Technology, Cambridge, MA, United States; ^4^Centre for the AIDS Programme of Research in South Africa (CAPRISA), Durban, South Africa; ^5^Department of Medical Microbiology, University of KwaZulu-Natal, Durban, South Africa; ^6^Medical Research Council - Centre for the AIDS Programme of Research in South Africa (MRC-CAPRISA) HIV-TB Pathogenesis and Treatment Research Unit, Doris Duke Medical Research Institute, University of KwaZulu-Natal, Durban, South Africa; ^7^Department of Virology, University of Bonn, Bonn, Germany; ^8^Department of Immunology and Infectious Diseases, Harvard T. H. Chan School of Public Health, Boston, MA, United States

**Keywords:** recurrent tuberculosis, antibodies, IgG3, *Mycobacterium tuberculosis*, recurrence

## Abstract

South Africa has the highest prevalence of HIV and tuberculosis (TB) co-infection globally. Recurrent TB, caused by relapse or reinfection, makes up the majority of TB cases in South Africa, and HIV infected individuals have a greater likelihood of developing recurrent TB. Given that TB remains a leading cause of death for HIV infected individuals, and correlates of TB recurrence protection/risk have yet to be defined, here we sought to understand the antibody associated mechanisms of recurrent TB by investigating the humoral response in a longitudinal cohort of HIV co-infected individuals previously treated for TB with and without recurrent disease during follow-up, in order to identify antibody correlates of protection between individuals who do not have recurrent TB and individuals who do. We used a high-throughput, “systems serology” approach to profile biophysical and functional characteristics of antibodies targeting antigens from *Mycobacterium tuberculosis (Mtb)*. Differences in antibody profiles were noted between individuals with and without recurrent TB, albeit these differences were largely observed close to the time of re-diagnosis. Individuals with recurrent TB had decreased *Mtb*-antigen specific IgG3 titers, but not other IgG subclasses or IgA, compared to control individuals. These data point to a potential role for *Mtb*-specific IgG3 responses as biomarkers or direct mediators of protective immunity against *Mtb* recurrence.

## Introduction

*Mycobacterium tuberculosis* (*Mtb*), which causes tuberculosis (TB), is the leading cause of death from an infectious agent of death in the world, with an estimated 10 million infections per year and 1.4 million deaths worldwide in 2019 (1). Moreover, TB is the leading cause of mortality among HIV-infected individuals (2); HIV-infected individuals have a possible increase in susceptibility to *Mtb*-infection, a greater likelihood to develop active TB disease, and higher risk of death from active TB (WHO 2018) (3). Past studies have suggested that HIV-infection correlates with increased risk of TB reinfection, making this population especially vulnerable for recurrent TB disease (4). Moreover, loss of CD4+ T cell help and global changes in mucosal immunity have been linked to HIV-associated susceptibility to *Mtb* infection and disease (5). However, the specific immunologic changes, and particular biomarkers, that identify individuals at greatest risk of active TB remain unclear; this could provide critical insights for patient management as well as point to unique immunological mechanisms that may contribute to *Mtb* control (6).

Recurrent TB in previously treated individuals constitutes 5-30% of the TB burden worldwide, occurring due to reactivation or reinfection with other *Mtb* strains (7). In the absence of HIV co-infection, patients successfully treated for TB have a reported 2-3% rate of recurrence, but this rate markedly increases in patients with HIV co-infection, especially after multiple TB episodes of treatment and recurrence (7, 8). In HIV co-infected individuals, 14% of initially cured patients may experience TB recurrence, with 88% of these reinfections occurring due to infection with a different/new *Mtb* strain (9). Currently, little is known about the immunologic factors that underlie the risk of recurrent TB, but increased levels of interleukin (IL) 6, IL-1β and IL-1Rα have been associated with increased rates of TB recurrence (10), suggesting that inflammatory responses might affect susceptibility to recurrent TB.

Emerging data point to a potential role for antibodies both as critical biomarkers of disease activity (11) and as intimate players in the anti-microbial response (12, 13). Specifically, changes in *Mtb*-specific antibody function, isotype distribution, and Fc-glycosylation have been linked to different *Mtb* disease states (14, 15), and distinguish between individuals with active and latent TB infection (16). Successful passive transfer of antibodies in TB has been observed with several monoclonal antibodies (17, 18) and polyclonal sera (19), arguing for a role for antibodies both as biomarkers of disease activity, but also as direct contributors to *Mtb* control. Additionally, a population of individuals, termed “resisters” that do not acquire TB infection despite confirmed continuous exposure, possesses unique antibody avidity and distinct *Mtb*-specific IgG Fc glycosylation profiles (14), raising the question of if there are similar antibody related protective mechanisms for individuals who do not get recurrent TB.

To begin to explore the potential role of specific Mtb antigen-specific antibodies in control of TB and control of progression to recurrent disease, we comprehensively profiled the *Mtb*-specific response in a South African cohort of HIV infected individuals previously cured from TB disease, confirmed by sputum smear microscopy, that were followed longitudinally for recurrent TB (20). We identified an enrichment of *Mtb*-specific IgG3 in individuals who did not develop recurrent TB, suggesting that higher IgG3 levels might play an important role in protection from reinfection.

## Methods

### Sample Cohort

Patients were enrolled in the “TB Recurrence upon Treatment with HAART” (TRuTH) study after completing treatment for drug-sensitive pulmonary TB in the CAPRISA SAPiT trial (10, 21–23) to determine the extent of and reasons for relapse and re-infection in incident cases of tuberculosis among HIV infected patients on ART, previously successfully treated for TB. Studies were conducted in South Africa at the Centre for the AIDS Programme of Research in South Africa (CAPRISA) in Durban, South Africa. HIV-infected patients on Highly Active Antiretroviral Therapy (HAART) diagnosed with TB were treated with the standard TB regimen, and outcomes were recorded as per the South African National TB control program guidelines. The study was conducted between June 2005 and August 2013 and patients were monitored for TB quarterly for 4 years, with clinical screening including chest radiograph and available TB diagnostic testing methods. The majority of patients did not have TB disease symptoms despite being diagnosed microbiologically with TB.

Specifically, we conducted a nested case control study where plasma samples from 34 individuals with (cases) and 38 individuals without (controls) recurrent *Mtb*-infection were included in this study with assessment of TB at study enrollment and 3-monthly thereafter. Cases and controls were matched for gender and timing of ART initiation in a 1:2 recurrent TB: no recurrent TB ratio. Ethical approval for the clinical study was obtained from the University of KwaZulu-Natal Biomedical Ethics Research Committee (BF051/09, NCT01539005). Written informed consent was obtained from all participants to publish case details and images with no personal identifiers.

### Antibody Subclass, Isotypes, and FcR Binding Analysis

In order to measure antigen-specific antibody subclasses, isotypes, and Fc-receptor (FcR) binding levels, a customized multiplexed Luminex assay was used, as previously described (24). This assay allows for relative quantification of antigen-specific humoral responses in a high-throughput manner using a Luminex MagPlex platform. A panel of antigens including PPD (Statens Serum Institute), ESAT-6/CFP-10 (BEI resources, NR-14868 and NR-49425), LAM (BEI resources, NR-14848), Hspx BEI resources, (NR-49428), Ag85AB complex (BEI resources, NR-14855), RV0826 and RV1363 (provided by Tom Ottenhoff, TBVI) was used.

In brief, antigens were coupled to individual fluorescent magnetic carboxyl-modified microspheres (MagPlex, Luminex Corporation) using 1-Ethyl-3- (3-dimethylaminopropyl) carbodiimide (EDC) (Thermo Fisher Scientific) and Sulfo-N-hydroxysuccinimide (NHS) (Thermo Fisher Scientific) per manufacturer’s instructions. The fluorescent and magnetic microspheres were activated using EDC and Sulfo in a buffer including NaH2PO4 for 30min at room temperature (RT), protein is added in a buffer containing 2-ethanesulfonic acid (MES) and were incubated for 2h at room temperature. Washing was then performed on a magnetic rack with PBS-Tween buffer post coupling. Subsequently, antigen-coupled microspheres were blocked with 1% bovine serum albumin (BSA), washed, and incubated for 16 hours at 4°C while rocking at 700 rpm with 1:10 diluted human plasma samples in PBS in a 384-well format to facilitate immune complex formation. The following day, plates were washed using an automated plate washer (Tecan Hydrospeed) with 0.1% BSA and 0.02% Tween-20. Antigen-specific antibody titers were detected with Phycoerythrin (PE)-coupled antibodies against IgG1, IgG2, IgG3, IgG4, IgA, and IgM (Southern Biotech). To measure antigen-specific Fc-receptor binding, Fc-receptors (FcR2AH, 2B, 3AV, and 3B, Duke Protein Production facility) were biotinylated with a BirA Kit (Avidity) according to manufacturer’s instructions. Biotinylated Fc-receptors were coupled to Streptavidin-PE (Prozyme) for 10min at room temperature and then added to immune-complexed beads to incubate for 1 hour at room temperature while shaking at 700 rpm. Fluorescence was detected using an Intellicyt iQue Screener plus cytometer utilizing a PAA robot arm. Analysis was performed *via* Forecyt software, graphing occurred using Prism 9 for MacOS (GraphPad). The readout was mean fluorescence intensity (MFI) of PE for each antigen coupled bead. All experiments were performed in duplicate while operators were blinded to study group assignment.

### Antibody-Dependent Cellular Phagocytosis

Antibody-dependent cellular phagocytosis (ADCP) was assessed in a bead-based assay (25). Antigens were biotinylated using Sulfo-NHS-LC-LC Biotin for 2h at room temperature (Thermo Scientific) and excess biotin was removed with a 3-kDa molecular mass cutoff column (Amicon/EMD, UFC500396). Yellow, fluorescent neutravidin beads (Thermo Fisher Scientific) were coupled to biotinylated ESAT-6/CFP-10, PPD and LAM for 2 h at 37°C. Subsequently, beads were washed and blocked with 1% BSA for 1h at room temperature. Then, antigen-coupled beads were incubated with 1:30 diluted plasma in PBS for 2 hours at 37°C. Following immune complex formation, samples were washed and 2.5x10^4^ THP-1 cells (ATCC) were added per well and incubated for 16 hours at 37°C in RPMI media with beta-mercaptoethanol. Following fixation, the next morning, sample acquisition was performed *via* flow cytometry (Intellicyt, iQue Screener plus) utilizing a robot arm (PAA), and analysis occurred using Forecyt software. Gating strategy included gating on THP-1 cells, single cells and FITC-positive events. A phagocytosis score was calculated as (percentage of bead-positive cells) x (MFI of bead-positive cells) divided by 10,000.

### Antibody-Dependent Neutrophil Phagocytosis

As described for the ADCP assay, biotinylated LAM, ESAT-6/CFP-10 and PPD were coupled to yellow fluorescent neutravidin beads (Invitrogen) (26). Plasma samples, diluted 1:30 in PBS were incubated with antigen-coupled beads for 2h at 37°C. Ammonium-Chloride-Potassium ACK lysis was performed on whole blood from healthy blood donors (MGH blood donor center) to isolate cells, and 5x10^4^ cells were added per well and incubated for 1 hour at 37°C. Subsequently, a PacBlue anti-CD66b detection antibody (clone G10F5) (RUO) (BioLegend) was used to stain for neutrophils for 15min at room temperature. Data acquisition occurred *via* flow cytometry (Intellicyt, iQue Screener plus) utilizing a robot arm (PAA), and analysis was performed using Forecyt software. Cells were gated on the cell population, single events, CD66b-positive neutrophils and FITC-positive neutrophils that phagocytosed fluorescent beads. A phagocytosis score was calculated as (percentage of bead-positive neutrophils) x (MFI of bead-positive neutrophils) divided by 10,000.

### Analysis

Data analysis was performed using Prism GraphPad (V9.0), R (4.0.2) and Python (3.9.0). Spearman correlation was performed for **Figure 2B** in GraphPad, Bonferroni correction was applied for multiple comparisons and adjusted p values are shown as asterisk, p value *< 0.05, **< 0.01, ***<0.001, ****<0.0001.

**Figure 1:**
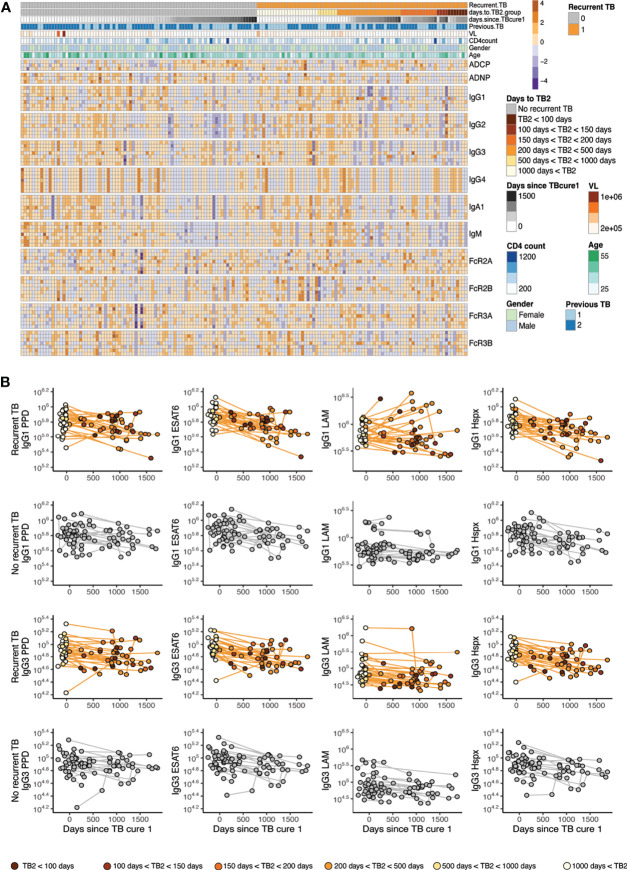
Mtb-specific antibody profile across recurrent/no recurrent TB patient cohort. **(A)** The annotated heatmap shows antibody measurements across time, groups and displays demographic data. The first row of the heatmap delivers information of grouping into individuals who go on to get recurrent TB (orange) versus who do not get recurrent TB (grey). Days to TB recurrence for the recurrence group is indicated in shades of orange, days from cure are colored in grey for all individuals. The row below in blue shades reveals if participants had (1) or did not (2) have previous TB before enrollment into the parent study. Age, gender, CD4 levels and viral load are indicated. Antibody effector functions, titer and Fc-receptor binding are displayed, antigens for the functions are PPD, EDAT6/CFP10 and LAM, additional antigens for titer and Fc-receptor binding include Hspx, Ag85, RV0826 and RV1363, in this order. **(B)** IgG1 and IgG3 titers are shown over time for the recurrent TB group (top) and the no recurrence group (bottom) for PPD, ESAT6/CFP10, LAM and Hspx. X axis depicts time since TB cure1 at enrollment into the study and color in the recurrence group represents time to TB recurrence in shades of orange.

**Figure 2:**
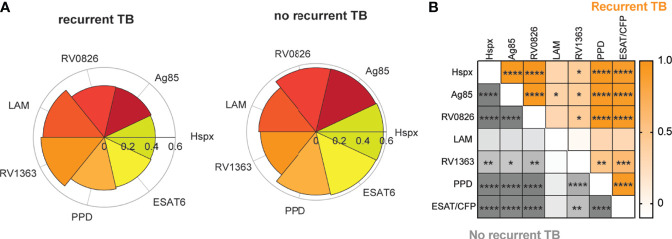
Mtb-specific antibody IgG1 titers correlate across different antigens. Graphs show the most distant time point from time since cure (and closest time point to recurrence for the recurrent TB group) for each individual in both groups. **(A)** Nightingale rose charts show comparisons between IgG1 titers against different TB-antigens between individuals with recurrent and no recurrent TB. Petals are scaled based on the median of the percent rank of each feature per group. **(B)** The Spearman correlation heatmap depicts correlation r values for the no recurrence group (grey) and the recurrence group (orange) for IgG1 titers across antigens. Color depth indicates r values, asterisk represent significance, adjusted for multiple comparison using Bonferroni correction method, adjusted p value *< 0.05, **< 0.01, ***<0.001, ****<0.0001.

**Figure 3:**
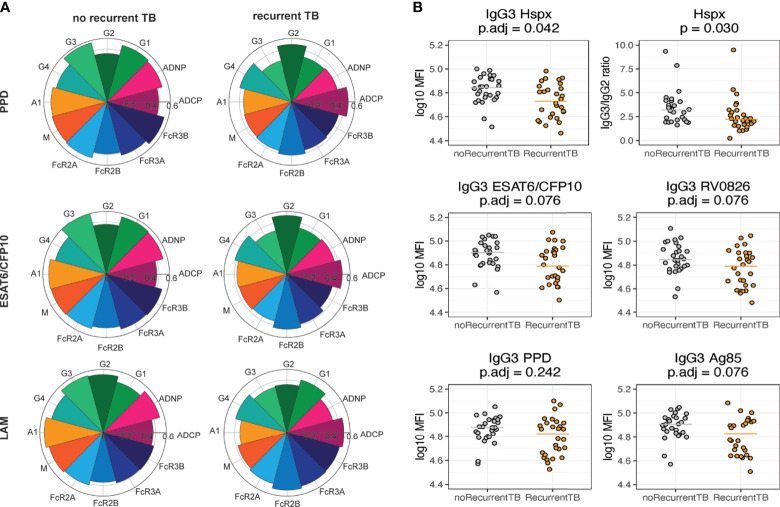
Individuals who do not get recurrent TB have higher titers of IgG3. All graphs show data for the most distant time point from time since cure. **(A)** Nightingale rose charts allow for comparison between groups for antibody titer (IgG1-IgG4, IgA, IgM), Fc-receptor binding levels (FCR2A, 2B, 3A, 3B) and the ability to induce effector functions (ADCP, ADNP) at the most distant time point from TB cure1. The left column shows flowers for the recurrent TB group, the right side for the no recurrence group. Relative responses for PPD (top), ESAT6/CFP10 (middle) and LAM (bottom) are shown. **(B)** Dot plots show univariate IgG3 levels (MFI) across different Mtb antigens, median is indicated by line. For Hspx, IgG3:IgG2 ratios are depicted additionally. Grey depicts no recurrence, orange recurrent TB. Each dot represents the average within each matching bin. P-values were calculated using Wilcoxon signed-rank test and adjusted for multiple comparison using Benjamini-Hochberg method.

The matching of cases and controls were taken into account for analyses comparing IgG3 (**Figure 3B**) and IgG3/IgG2 ratio for Hspx (**Figure 3B**) in individuals with recurrent TB vs individuals with no recurrent TB. For these analyses, data points from the most distant time point from TB cure1 were used. In **Figure 3B** the matching bin averaged values were visualized, and p-values from the Wilcoxon signed rank test were corrected for multiple comparison using Benjamini-Hochberg method and the adjusted values are reported on top of each graph.

**Figure 4:**
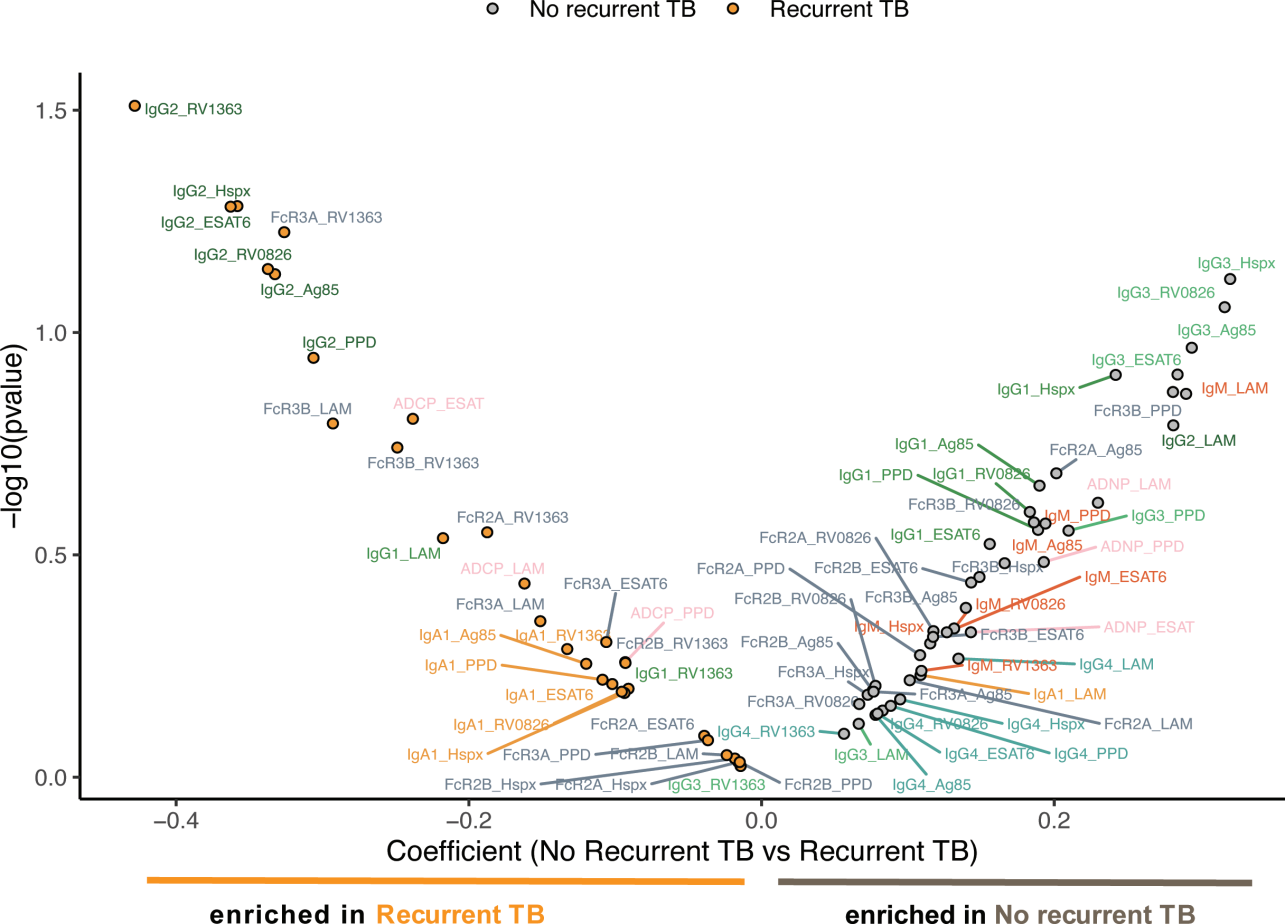
Mixed effects model supports the indication of higher IgG3 levels in no recurrent TB individuals. Dot plots and the model depict the most distant time point from time since cure for every individual. The mixed effects models depict the overall differences in measured antibody features between individuals with (left side) and without (right side) recurrent TB. The models include recurrent and non-recurrent individuals taking into account age, gender, as well as days since TB cure1. The X-axis depicts the effect size between the groups and y-axis shows a measure of statistical significance.

For linear mixed-effects models R package *lme4* (27) was used (**Figure 4**). Immunological data were standardized (z-scored) to achieve a mean of zero and a standard deviation of one. Linear mixed-effects models with random effects of the individual participant were built, where each measured antibody feature was analyzed separately as the response variable. The compound symmetry structure was used to account for correlation between measurements from the same participant. For each feature, variable intercept null and full models were fit using the subject ID. Null models consist of sex, age, and time since TB cure 1, the full models include the group effect (recurrent TB, no recurrent TB). A likelihood ratio test was applied using the null and full models to obtain the significance of the group effect as p-values. These p-values were then plotted against the coefficient of the group effect (β values) in **Figure 4B**.

## Results

### Heterogeneous Antibody Responses Are Observed Across the Cohort

While antibodies have been implicated as biomarker of TB disease (11), it remains unclear whether changes in *Mtb*-specific antibodies also mark changes in susceptibility to TB disease, heralding loss of TB control and progression to disease. Thus, we comprehensively characterized the humoral immune response across time in a group of individuals with or without recurrent TB. Specifically, the biophysical and functional *Mtb*-specific humoral response was profiled in human plasma from the TRuTH study (21), including 34 individuals that experienced TB recurrence and 38 individuals that did not re-experience TB over the same 4 year follow up period.

A customized multiplex Luminex assay was utilized to measure antibody isotype and subclass responses and the ability of antigen-specific antibodies to bind to Fc-receptors. These features were measured against a variety of *Mtb*-specific antigens, including lipoarabinomannan (LAM), purified protein derivative (PPD), the 6kDa sized early secretory antigenic target (ESAT6) in combination with the culture filtrate protein 10 (CFP10), heat shock protein HspX, and two *Mtb* membrane proteins, RV0826 and RV1363 (28). Additionally, we performed *in vitro* functional assays of antibody-dependent cellular phagocytosis and antibody-dependent neutrophil phagocytosis against PPD, LAM, and ESAT6/CFP10.

To gain an overall picture of the humoral immune profiles across the groups, a heatmap was constructed including all humoral features across recurrent and non-recurrent individuals, as well as across the time course of the cohort (**Figure 1A**). Each column represents an individual, the top rows indicate cohort information such as time since TB cure1 and time to TB2 (Tb recurrence time point), followed by antibody measurements, ordered by feature and antigen. These data highlight the heterogeneity in humoral profiles across the two groups and over time, beginning to point to unique patterns across the groups. Specifically, blocks of low-level *Mtb*-specific IgG2s were noted in the individuals without recurrent TB. Conversely, lower levels of *Mtb*-specific IgG3 immunity were noted in individuals with recurrent TB. Thus, these data begin to suggest different trends across time and groups regarding recurrent *Mtb* infection.

### IgG1 Responses Decline Following Cure of TB1 Against PPD and ESAT6/CFP10

To further analyze the longitudinal aspect of the cohort, we examined the longitudinal changes in PPD-, ESAT6/CFP10-, LAM-, Hspx-specific IgG1 and IgG3 antibody responses over time across the groups. To account for variation in sampling times across the groups, all individuals were aligned to days since initial cure from TB infection (Cure1) (**Figure 1B**). Similar declines in *Mtb*-specific antibody titers were noted across the two groups following initial cure of TB infection (TB cure1, confirmed by negative sputum testing) or day 0 across IgG1 and IgG3. However, a fraction of individuals in the recurrent group demonstrates an increase in IgG1 but not IgG3 titers against *Mtb*-specific antigens, most notably against the mycobacterial cell wall antigen LAM. Whether this increased LAM-specific immunity reflects persistent exposure in a fraction of individuals or recapitulates previous observations of increasing LAM-specific antibodies following successful treatment of active TB (29) remains unclear. However, this observed increase in LAM-specific immunity, notably, was only observed in individuals with subsequent TB-recurrence.

### Antibody IgG1 Titers Are Highly Correlated Across Antigens, Except for RV1363 and LAM

To gain deeper insights into the specific differences across individuals that ultimately developed recurrent TB compared to those that didn’t, we examined *Mtb*-specific humoral immune responses at the most distant time point since TB Cure1 for each of the individuals. This allowed for a comparison of the closest time point to TB2 for the recurrent group versus similar follow-up time points for the group without recurrent TB. Antibody profiles were interrogated across a range of antigens including Hspx (30), Ag85 (31), and the membrane proteins RV0826 and RV1363 (28) (**Figure 2A**).

Trends towards increased median IgG1 responses to PPD, ESAT6/CFP10, Hspx, Ag85, and RV0826 in individuals with no-recurrent TB at the last timepoint prior to TB in the TB reinfection group can be observed. Conversely, LAM- and RV1363-specific IgG1 responses appear to be expanded in individuals with recurrent TB. These data point to differences in the immunogenicity of particular *Mtb* antigens. Similar trends among certain antigenic targets suggests a connection and synergy between these antigens. Therefore, we aimed at analyzing the correlation between IgG1 responses across various antigens. Analysis of the relationships across antigen-specific antibody responses at the final timepoint across both groups revealed strong IgG1 coordination against different *Mtb-*specific antigens within two study groups (**Figure 2B**). However, slightly increased relationships were noted between RV1363 and PPD in individuals without recurrent TB, pointing to differences in coordination across antigens. However, whether the same relationships also exist among HIV uninfected or untreated cohorts remains unknown.

### *Mtb*-Specific IgG3 Is Increased in Individuals Who Do Not Develop Recurrent TB

In order to further investigate antibody feature differences that differed across the two groups, median antibody titers, functions and Fc-receptor binding activity were compared for PPD-, LAM-, and ESAT6/CFP10-specific responses (**Figure 3A** **)**. These 3 antigens were chosen as PPD is composed of a variety of different proteins, ESAT6/CFP10 antigens are known to be *Mtb*-specific, and LAM is a glycan antigen, allowing to capture responses targeted towards a variety of antigen types. Robust responses were observed across the two groups, however, the nightingale plots appeared larger in the individuals without recurring TB across antigens. Specific differences began to emerge including higher levels of IgG2 to PPD and ESAT6/CFP10 in individuals with recurrent TB and the presence of higher levels of IgG3 across all three antigens in individuals who did not develop recurrent TB (**Figure 3A**). These data point to the potential maintenance of poorly functional IgG2 antibody subclasses in the context of HIV and *Mtb* co-infection in individuals that are more likely to become reinfected and highly functional IgG3 antibodies in individuals that resist TB reinfection (32). Accordingly, individuals without recurrent TB exhibited enhanced neutrophil phagocytosis (ADNP). In contrast, IgG4, another less functional IgG subclass, and inhibitory FcR2B-binding were slightly higher in the group with recurrent TB across antigens. Importantly, IgG3 titers were not only elevated against these three antigens in individuals without recurrent TB, but also against other *Mtb*-specific antigens such as Hspx, RV0826 and Ag85 (**Figure 3B**). Interestingly, no evident difference in IgG3 levels in individuals with multiple episodes of TB versus individuals with only one episode was observed, suggesting that multiple recurrences did not affect IgG3 levels. Moreover, a disconnect between antibody isotypes and subclasses in individuals with recurrent TB was observed, with less coordination between IgG3, IgG4 and IgM responses (**Supplementary Figure 1**), suggesting differential isotype/subclass selection profiles across the groups.

Since IgG3 and IgG2 levels were different between the groups and differences in IgG3:IgG2 ratios have been observed to be important for protection in other diseases such as HIV (33), here we analyzed IgG3:IgG2 ratios in individuals with and without recurrent TB, only ratios for Hspx are shown due to the significant differences between the two groups. When comparing ratios of IgG3 to IgG2 levels for Hspx between the two groups, individuals who experienced recurrent TB had higher IgG3:IgG2 ratios (**Figure 3B**), pointing to a consistent and striking deployment of distinct IgG subclasses across the infected groups.

To ultimately define the multivariate signatures associated with TB recurrence, we finally applied a linear mixed effect model to define the features that differed across the groups, while balancing for age, gender and time since TB cure 1 (**Figure 4**). The model highlighted the enrichment of IgG3 features in the individuals that did not experience recurrent TB, while a clear enrichment for IgG2-specific *Mtb* responses were observed in individuals with recurrent TB. While only IgG3 titers against Hspx were significantly different close to recurrence (**Figure 3B**), an enrichment of IgG3 responses was also observed across multiple additional antigens, including RV0826, Ag85, and ESAT6/CFP10 in the mixed effect model. Thus, these data argue for significant persistent *Mtb*-specific subclass selection differences over time, independent of demographics, as differentiators of susceptibility to TB reinfection. Given our emerging appreciation for the antimicrobial role of antibodies, these data collectively suggest that the maintenance of distinctly functional *Mtb*-specific IgG subclass responses may predict differential risk to TB reinfection.

## Discussion

TB is the leading infectious disease killer across the globe, with elevated mortality rates among HIV-infected populations (34). The high prevalence of recurrent infections with *Mtb* in HIV-infected individuals continues to place a significant burden on the healthcare system, especially in high incidence countries, such as South Africa. However, why TB reinfection occurs in a fraction of those previously infected remains unclear. Previous studies aimed at profiling differences in cytokine responses of individuals with recurrent disease (10), applied whole genome sequencing (35) and whole blood transcriptomic analysis (20), finding IL-1Rα and a transcriptomic correlate of risk (COR) signature as markers of risk for recurrent TB. Given our emerging appreciation for the role of antibodies as both biomarkers and potential contributors to antimicrobial immunity (36), analysis of the humoral profile in regard to recurrent TB infection can help decipher the underlying mechanisms of recurrent TB disease susceptibility.

In order to lower the rate of recurrent infections, it is critical to identify the correlates of protection in individuals who do not develop recurrent TB. Here, we have conducted the first in-depth analysis of *Mtb*-specific humoral responses in the context of TB recurrence when co-infected with HIV-1. Through a comprehensive serological profiling of a longitudinal cohort of HIV/TB co-infected individuals, a community with increased vulnerability to recurrent infection, we observed variable antibody titer levels against *Mtb* antigens within the cohort. While individuals who did re-experience TB exhibited a trend of overall declining antibody IgG1 and IgG3 titers since time of TB cure, these individuals possessed overall lower IgG3 titers across multiple antigens compared to individuals that experienced reinfection/reactivation. The lower levels of IgG3 in the recurrent TB group were only detected at a time point close to TB recurrence, indicating that IgG3 might play a protective role or mark certain immunological changes proximal to the timepoint of recurrence.

In general, a decrease in antibody titers over time since cure was observed across groups and protein antigens, consistent with successful pathogen clearance (37). Similar observations have been made in malaria (38), HIV-1 (39) and influenza (40). Recent studies have shown that TB treatment is associated with declining levels of ESAT6-specific antibody levels, with a concomitant rise in LAM-specific titers (41), pointing to diverging antibody profile evolution across antigen-specificities. We observed similar trends of declining ESAT6/CFP10- and PPD-specific IgG1 antibodies post treatment, while some individuals exhibited a spike in their LAM-specific IgG1 responses over time, suggesting either an exposure- or treatment-related antibody expansion (42). Previous studies have implied *Mtb*-specific IgG as a marker for active disease, suggesting that the increase in IgG1 against LAM may also mark a recent exposure or a sign of disease onset (11, 43), and potentially, recurrent TB could cause changes to the immunological profile much earlier than clinical diagnosis. However, whether the increase in LAM-specific antibodies is a marker of a renewed antibody response in a subset of individuals or a novel exposure in this high exposure setting remains unclear, but the data point to diverging overall antibody trajectories across the population in this cohort. In the future, it will be critical to evaluate similar responses in HIV-uninfected individuals, as well as HIV-coinfected individuals off therapy, to decipher if the same humoral biomarkers track with TB recurrence.

While IgG1 antibody levels were similar in individuals with and without recurrent TB, IgG3 responses to protein antigens were enriched among individuals who did not go on to get recurrent TB. IgG3 is the first IgG in the IgH locus, representing the first IgG subclass selected after an acute infection or immune response (44). In this regard, IgG3 responses are typically induced early upon antigen exposure or infection (32) and are known to induce high levels of antibody-mediated effector functions, due to the higher affinity of the IgG3 Fc-domain to Fc-receptors (45). In some instances, IgG3 responses can be preserved in immunological memory, locked into the humoral immune response to continue to provide highly functional immunity against a pathogen. Along these lines, individuals that resist malaria infection appear to produce long-lived IgG3-responses that enable them to overcome yearly exposures to the parasite (46), likely through the rapid deployment Fc-effector functions, key to the control and elimination of the parasite. IgG3 is known to activate especially NK cell related functions very efficiently (47), and given the emerging appreciation for a role of NK cells, and potentially antibody dependent cellular cytotoxicity (15), in TB infection (48), these data may point to a functional role of *Mtb*-specific IgG3 antibodies in anti-microbial control that may contribute to protection against recurrent TB due to NK cell induced functionality.

Beyond the elevation of IgG3, individuals without recurrence also tended to possess slightly lower levels of IgG2 and IgG4. IgG2 and IgG4 have the lowest affinity for Fc-receptors in humans (49), pointing to the evolution of less functional IgG subclasses among individuals that are vulnerable to recurrence (50). Furthermore, IgG3:IgG2 ratio for Hspx differed significantly between the groups close to the time of TB recurrence. Interestingly, the same subclass imbalance has been linked to compromised responses to HIV (33) and malaria (51). Whether these antibodies contribute directly to control of *Mtb* remains unclear, but they could also mark the presence of distinct T-cell helper profiles that may contribute differentially to microbial control deep within the lungs. Therefore, investigating the T cell responses in HIV-infected or treated individuals over time may provide critical insights on the direct or indirect anti-microbial mechanisms involved in preventing recurrent TB However, critically, a growing field of emerging work suggests that antibodies may not simply be markers of disease but can also contribute to antimicrobial activity.

The data presented here suggest that significant changes in the humoral immune response may occur with the resolution of infection and may point to specific immunological mechanisms that may be key to persistent protection from infection in this cohort. Whether *Mtb*-specific IgG3 can prevent progression or directly contribute to the resolution of disease or these differences in titers present altered reactivity in the face of simmering disease in recurrent individuals, as well as whether it can be induced by vaccines, remains unclear. Also, the role of HIV in these observed responses remains elusive and warrants further investigation. However, this work bolsters antibodies as providing novel immunological opportunities to leverage protective immunity to *Mtb*, in order to understand disease biology and advance vaccine and therapeutic design.

## Data Availability Statement

The raw data supporting the conclusions of this article will be made available by the authors, without undue reservation.

## Ethics Statement

The studies involving human participants were reviewed and approved by University of KwaZulu-Natal Biomedical Ethics Research Committee (BF051/09, NCT01539005). The patients/participants provided their written informed consent to participate in this study.

## Author Contributions 

SF, KN and GA designed the research study. SF and SS conducted the experiments and acquired the data. SF and DC analyzed and visualized the data under guidance of DL, AS, KN and NY-Z provided study samples. SF, DC and GA wrote the manuscript. HS, AS, KN, NY-Z, LD, SMF and PG gave valuable comments and input on the manuscript. All authors contributed to the article and approved the submitted version.

## Funding

This work was supported by the National Institutes of Health under award number R56-AI155149, R37-AI080289, R01-Al124348, the Ragon Institute Sundry and the Gates Foundation (OPP1151840). The TRUTH study was supported by the Howard Hughes Medical Institute, Grant Number 55007065, as well as the Centers for Disease Control and Prevention (CDC) Cooperative Agreement Number UY2G/PS001350-02.

## Author Disclaimer

Its contents are solely the responsibility of the authors and do not necessarily represent the official views of either the Howard Hughes Medical Institute or the Centers for Disease Control and Prevention (CDC).

## Conflict of Interest

The authors declare that the research was conducted in the absence of any commercial or financial relationships that could be construed as a potential conflict of interest.

## Publisher’s Note

All claims expressed in this article are solely those of the authors and do not necessarily represent those of their affiliated organizations, or those of the publisher, the editors and the reviewers. Any product that may be evaluated in this article, or claim that may be made by its manufacturer, is not guaranteed or endorsed by the publisher.

## Supplementary Material

Supplementary Figure 1 The Spearman correlation heatmaps each show the correlation between different isotypes and subclasses for one antigen and either for individuals who do or do not get recurrent TB. The color indicates the correlation r value, which are also displayed on the graph. Correlations are corrected for multiple comparisons.
