# Supplementary figures and images for: A Mycobacterium tuberculosis Specific IgG3 Signature of Recurrent Tuberculosis
Source: Front Immunol. 2021 Sep 22;12:729186. doi: 10.3389/fimmu.2021.729186 (PMC8493041; doi:10.3389/fimmu.2021.729186)

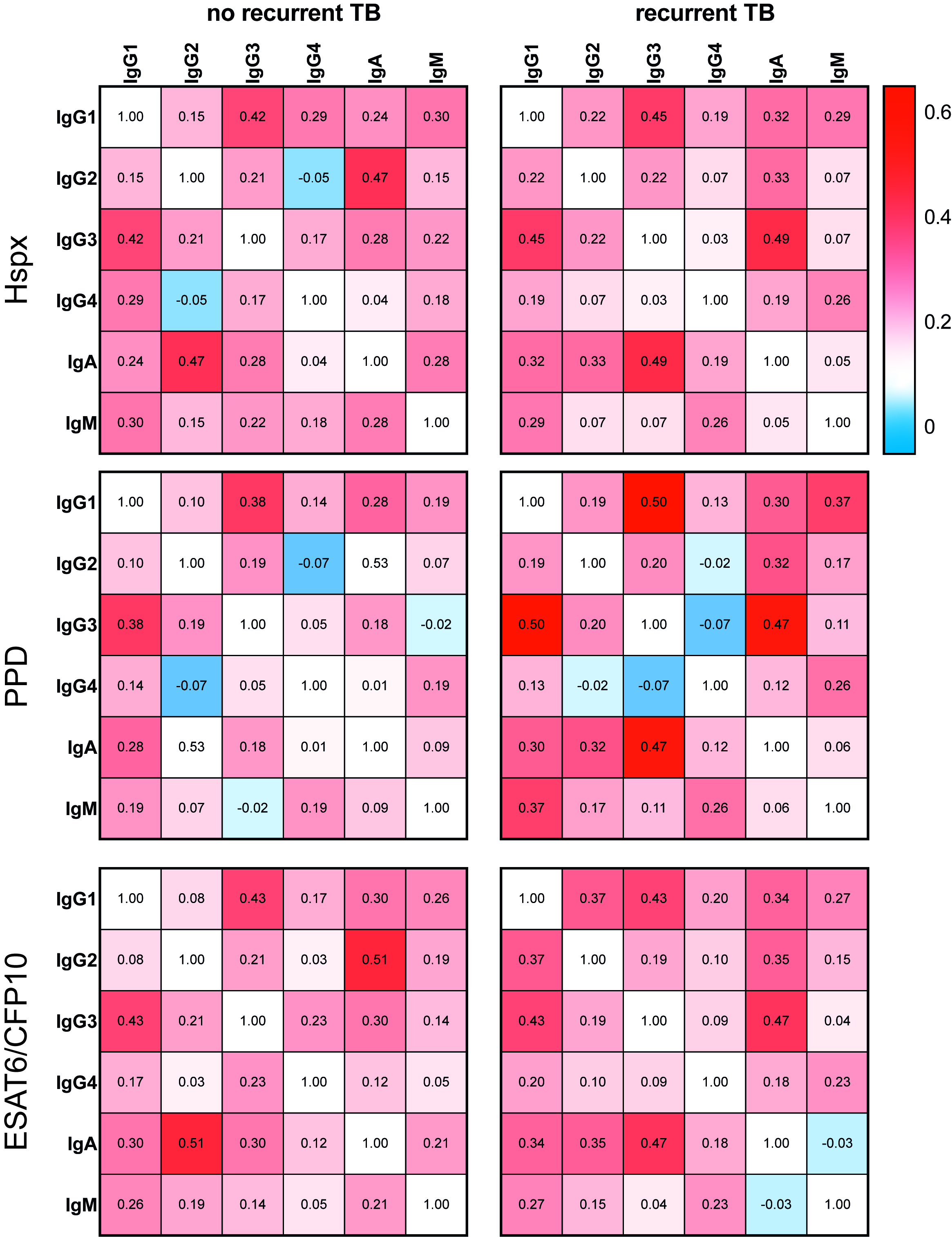

Supplement: Supplementary Figure 1 — The Spearman correlation heatmaps each show the correlation between different isotypes and subclasses for one antigen and either for individuals who do or do not get recurrent TB. The color indicates the correlation r value, which are also displayed on the graph. Correlations are corrected for multiple comparisons. [file Image_1.tiff]
